# Small RNA discovery in the interaction between barley and the powdery mildew pathogen

**DOI:** 10.1186/s12864-019-5947-z

**Published:** 2019-07-25

**Authors:** Matt Hunt, Sagnik Banerjee, Priyanka Surana, Meiling Liu, Greg Fuerst, Sandra Mathioni, Blake C. Meyers, Dan Nettleton, Roger P. Wise

**Affiliations:** 1 Interdepartmental Genetics & Genomics, Iowa State University, Ames, Iowa 50011 USA; 2 Department of Plant Pathology & Microbiology, Iowa State University, Ames, Iowa 50011 USA; 3 Interdepartmental Bioinformatics & Computational Biology, Iowa State University, Ames, Iowa 50011 USA; 4 Department of Statistics, Iowa State University, Ames, Iowa 50011 USA; 5 Corn Insects and Crop Genetics Research, USDA-Agricultural Research Service, Iowa State University, Ames, Iowa 50011 USA; 6 Donald Danforth Plant Science Center, St. Louis, MO 63132 USA; 7 Division of Plant Sciences, University of Missouri – Columbia, 52 Agriculture Lab, Columbia, MO 65211 USA

**Keywords:** *Blumeria*, Barley, Small RNA-Seq, Transposable elements, EKA family, CSEPs, Pathogen effectors

## Abstract

**Background:**

Plants encounter pathogenic and non-pathogenic microorganisms on a nearly constant basis. Small RNAs such as siRNAs and miRNAs/milRNAs influence pathogen virulence and host defense responses. We exploited the biotrophic interaction between the powdery mildew fungus, *Blumeria graminis* f. sp. *hordei* (*Bgh*), and its diploid host plant, barley (*Hordeum vulgare*) to explore fungal and plant sRNAs expressed during *Bgh* infection of barley leaf epidermal cells.

**Results:**

RNA was isolated from four fast-neutron immune-signaling mutants and their progenitor over a time course representing key stages of *Bgh* infection, including appressorium formation, penetration of epidermal cells, and development of haustorial feeding structures. The Cereal Introduction (CI) 16151 progenitor carries the resistance allele *Mla6*, while *Bgh* isolate 5874 harbors the *AVR*_*a6*_ avirulence effector, resulting in an incompatible interaction. Parallel Analysis of RNA Ends (PARE) was used to verify sRNAs with likely transcript targets in both barley and *Bgh. Bgh* sRNAs are predicted to regulate effectors, metabolic genes, and translation-related genes. Barley sRNAs are predicted to influence the accumulation of transcripts that encode auxin response factors, NAC transcription factors, homeodomain transcription factors, and several splicing factors. We also identified phasing small interfering RNAs (phasiRNAs) in barley that overlap transcripts that encode receptor-like kinases (RLKs) and nucleotide-binding, leucine-rich domain proteins (NLRs).

**Conclusions:**

These data suggest that *Bgh* sRNAs regulate gene expression in metabolism, translation-related, and pathogen effectors. PARE-validated targets of predicted *Bgh* milRNAs include both EKA (effectors homologous to *AVR*_*k1*_ and *AVR*_*a10*_) and CSEP (candidate secreted effector protein) families. We also identified barley phasiRNAs and miRNAs in response to *Bgh* infection. These include phasiRNA loci that overlap with a significant proportion of receptor-like kinases, suggesting an additional sRNA control mechanism may be active in barley leaves as opposed to predominant *R*-gene phasiRNA overlap in many eudicots. In addition, we identified conserved miRNAs, novel miRNA candidates, and barley genome mapped sRNAs that have PARE validated transcript targets in barley. The miRNA target transcripts are enriched in transcription factors, signaling-related proteins, and photosynthesis-related proteins. Together these results suggest both barley and *Bgh* control metabolism and infection-related responses via the specific accumulation and targeting of genes via sRNAs.

**Electronic supplementary material:**

The online version of this article (10.1186/s12864-019-5947-z) contains supplementary material, which is available to authorized users.

## Background

To prevent infection from potential pathogens, plants employ an integrated multi-phasic system, including a non-species-specific defense response tailored to pathogen type, and a pathogen-species specific response. For the first phase, plants perceive pathogen-associated molecular patterns (PAMPs) associated with pathogen type, such as chitin for fungi and flagellin for bacteria [1]. These molecules bind to receptor-like kinases to trigger PAMP-triggered immunity (PTI) that can include accumulation of cell wall material, a reactive oxygen species (ROS) response, and accumulation of antimicrobial compounds and hydrolytic enzymes [2]. Successful pathogens have evolved effector molecules that act to compromise the PTI response and lead to effector-triggered susceptibility (ETS). To combat pathogen effectors, plants evolved an additional response, designated effector-triggered immunity (ETI). ETI is the result of the interaction of resistance (R) proteins, often encoded by nucleotide-binding oligomerization domain (NOD)-like receptors (NLRs), and pathogen effector molecules [3, 4]. This interaction triggers a strong immune response, commonly associated with the hypersensitive reaction and localized cell death.

*Blumeria graminis* f. sp. *hordei* (*Bgh*) is an obligate biotrophic fungus of the phylum Ascomycota. Obligate biotrophic fungi complete their life cycle in living hosts, which requires the fungus to suppress host defense mechanisms and also to extract nutrients to support colonization. To accomplish these functions, obligate biotrophs express effector molecules that act both inside and outside host cells. Effectors actively suppress host defenses and create an environment conducive to fungal growth and reproduction. The *Bgh* genome is predicted to encode two different classes of effector proteins. The first class is designated EKA (effectors homologous to *AVR*_*k1*_ and *AVR*_*a10*_) that lack traditional targeting sequences for secretion and originate from Class I LINE retrotransposons [5]. The class name EKA comes from its two founding members AVR_K1_ and AVR_A10_ which were identified as targets of the barley R proteins MLK1 and MLA10 [6]. The second class, candidate secreted effector proteins (or CSEPs), were identified using several criteria, including presence of a predicted signal peptide for secretion, smaller size, and lack of homology to other known proteins outside of powdery mildews [7–9]. The two classes of effectors combined, represent around 2000 members, which is a substantial portion of the ~ 7000 protein-encoding genes [5, 10].

Expression of defense-related genes in plants and virulence genes in pathogens are often regulated at the post-transcriptional level by small RNAs (sRNAs). In most cases sRNAs function within the organism to regulate gene expression in an endogenous fashion. In plants, defense genes related to both PTI and ETI responses are regulated by micro RNAs (miRNAs) [11, 12]. Several miRNA families are involved in regulating plant responses to pathogen infection [13, 14]. The targets of these miRNAs are involved in both PTI and ETI responses. The PTI-related pathways regulated through miRNAs include hormone signaling, reactive oxygen species evolution, callose deposition, and others [11]. Auxin signaling is carefully controlled during plant development and can be down regulated during pathogen infection such as with miR393 that downregulates auxin F-box receptors during a PTI response to infection [15]. Callose deposition related to PTI response has both positive regulators such as miR160 and negative regulators such as miR398 and miR773 [13]. The ETI pathway is regulated through miRNA control of *R*-gene expression. MicroRNAs from several species including *Medicago truncatula*, soybean, tomato, potato, and tobacco have been shown to regulate *R*-gene expression [12]. The regulation of these *R*-gene-encoded transcript targets through miRNAs does not however, lead to simple transcript cleavage in many cases. Rather, the cleaved transcripts are targets for production of phased small interfering RNAs (phasiRNAs). These phasiRNAs can lead to silencing of hundreds of *R*-gene transcripts [16].

The occurrence of phasiRNAs was first observed in *Arabidopsis* with a type of phasiRNA called *trans*-acting small interfering RNAs (tasiRNAs) [17]. Unlike most phasiRNAs, tasiRNAs are usually encoded on long non-coding RNA templates. The miRNA-cleaved precursors are reverse transcribed into double stranded RNA by an RNA-dependent RNA polymerase (RDRP) and cleaved into 21-nt small RNAs that are produced in a regular or “phased” pattern, hence the name “phasiRNAs”. Four families of *TRANS ACTING siRNA* (TAS) genes have been identified in *Arabidopsis* including *TAS1*, *TAS2*, *TAS3*, and *TAS4* [18]. The resulting phasiRNAs then act in *trans* against targets including transcripts encoding auxin response factors, pentatricopeptide repeat proteins, and MYB transcription factors [19–21]. *TAS3* is the most highly conserved member of the TAS family and is found in plant species ranging from mosses, gymnosperms, to grasses [20, 22]. Grasses have a much larger set of tasiRNAs than found in eudicots [23]. These tasiRNAs are largely encoded on long non-coding transcripts expressed in reproductive tissues, and are 24 bases in length as opposed to eudicot phasiRNAs, which are typically 21 bases in length. Very few phasing loci have been reported in non-reproductive tissues in monocots, with few exceptions [24].

Filamentous plant pathogens have been shown to regulate virulence-related genes through the accumulation and deployment of sRNAs. In the oomycete pathogen *Phytophthora sojae* the avirulence factor Avr3a is differentially silenced by small RNAs in a transgenerational fashion, allowing for infection of plants with an R-protein recognizing the Avr3a protein [25]. In *Phytophthora infestans* sRNAs were identified that target numerous *RxLR* and *Crinkler* effector genes that were differentially accumulated between highly and weakly pathogenic strains [26]. Small RNAs of the microRNA-like (milRNA) type were differentially expressed in the plant fungal pathogen *Fusarium oxysporum* f. sp. *niveum* regulating the expression of the two toxins trichothecene and NEP1 [27]. Recently, sRNAs have been implicated in barley powdery mildew interactions as well [14]**.** Thus, tuning gene expression to influence resistance proteins and pathogenicity factors is clearly important for determining the outcome of plant/pathogen interactions.

In this study, we sought to identify sRNAs involved in the regulation of plant and fungal gene expression during *Bgh* parasitism of its barley host. To accomplish this goal we infected seedlings from barley line CI 16151 (containing the *Mla6* powdery mildew resistance allele) and four fast-neutron-derived immune-signaling mutants representing both compatible (*mla6*, *rar3*, and *mla6* + *bln1*) and incompatible (*bln1*) interactions. *Mla6* is a major NLR-type resistance gene [28, 29], while *Rar3* (*Required for Mla6 resistance* *3*) is an unlinked locus required for *Mla6* function. *Blufensin1* (*Bln1*) is an inhibitor of basal defense [28] and silencing of *Bln1* results in down-regulation of genes involved in nuclear import and the secretory pathway [30]. The *mla6* + *bln1* double mutant is susceptible (due to the *mla6* deletion)*,* but the *bln1* phenotype is masked in plants that contain the *Mla6 R* gene. The deletion mutants *mla6* or *rar3* are susceptible to *Bgh* 5874 infection, as opposed to the resistant CI 16151 progenitor, or *bln1*. RNA extracted from a 48-h time-course representing key stages of *Bgh* development (appressorium formation, penetration of epidermal cells, and development of haustoria) was used for whole transcriptome sequencing (RNA-Seq), small RNA sequencing (sRNA-Seq), and Parallel Analysis of RNA Ends (PARE) to identify barley and *Bgh* sRNAs and their transcript target sites. We present a catalogue of sRNAs predicted to target genes involved in metabolic processes and immune functions in both host and pathogen.

## Results

### Identification of Bgh and barley sRNAs

**Fig. 1:**
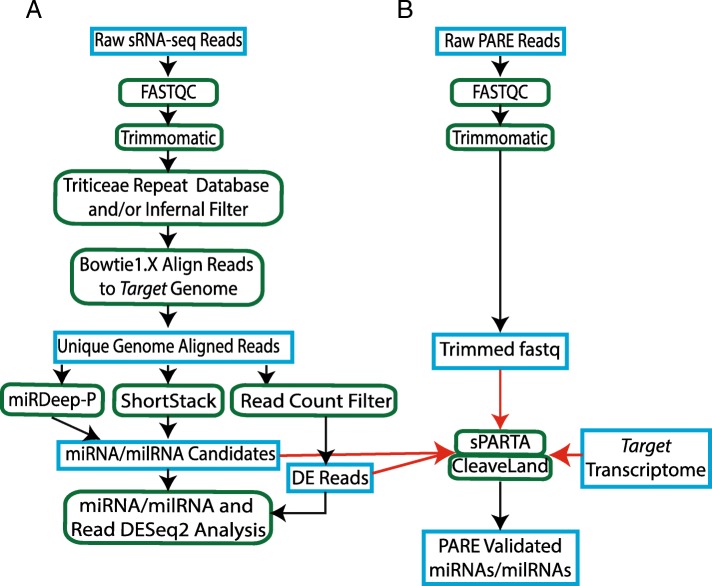
Small RNA sequencing and PARE sequencing analysis pipelines. (**A**) Small RNA-Seq Illumina reads were trimmed, filtered, and run through the two plant miRNA identification programs miRDeep-P and ShortStack to identify miRNA/milRNA candidates and DE reads. (**B**) Sequencing reads from the PARE libraries were trimmed and filtered and analyzed with the sPARTA version 1.21 [31] and CleaveLand (version 4.4) [32]. Additional input data was provided from the barley or *Blumeria* transcriptome and miRNA/milRNA candidates plus DE reads developed from the sRNA sequencing pipeline. Input or output files are highlighted with blue boxes, programs or processes are highlighted with green ovals, and PARE program inputs are highlighted with red arrows

To identify sRNAs in barley and *Bgh*, sRNA-Seq libraries were produced from barley line CI 16151 and four fast-neutron derived immune-signaling mutants infected with *Bgh* isolate 5874 (*AVR*_*a6*_). These lines include the susceptible mutants *mla6, rar3,* and *mla6* + *bln1*, as well as the resistant mutant *bln1*. *Bgh*-inoculated 1st leaves (5 genotypes × 6 time points × 3 biological replications) were harvested from a split-plot design at 0, 16, 20, 24, 32, and 48 HAI for a total of 90 samples. The sequenced libraries contained ~ 2.8 billion total reads that were filtered and mapped separately to the barley and *Bgh* genomes, as detailed in Fig. 1a. Because there are few, if any, fungal-specific resources for predicting functional sRNAs from sRNA sequencing data, we used the same two approaches for both the barley mapped reads and *Bgh* mapped reads. The first approach was to use two plant-specific miRNA prediction programs (ShortStack and miRDeep-P) to predict miRNAs/milRNAs with structural similarities to plant miRNAs from the barley and *Bgh* aligned reads [33, 34]. The ShortStack and miRDeep-P programs predicted a total of 1,425 barley miRNA candidates and 1,741 *Bgh* milRNAs candidates with plant miRNA-like structural features. The second approach was to filter reads with exact matches to the barley or *Bgh* genomes and with at least 10 counts across the 90 libraries. The reads that passed the mapping and count filters were designated either barley or *Bgh* genome mapped sRNAs. Applying this minimum abundance cutoff of 10 to the ~ 86 million unique reads from the full sRNA-Seq dataset, ~ 1.98 million reads mapped exactly to the barley genome and ~ 955,000 mapped exactly to the *Bgh* genome.

### Candidate Bgh milRNAs and genome mapped sRNAs are primarily differentially expressed at 48 HAI

To identify sRNAs important in *Bgh* development and successful barley infection, milRNA candidates and *Bgh* genome mapped sRNAs were analyzed for differential expression (DE) using the DESeq2 program [35]. The DESeq2 outputs were filtered for adjusted *p*-values of less than 0.05. Small RNA accumulation was analyzed at each time point, comparing *Bgh* sRNAs in the four mutant lines to those from the progenitor CI 16151. In total, 13311 (14.1%) of the *Bgh* genome mapped sRNAs and 268 (15.4%) of the milRNA candidates were DE in at least one time point as compared with wild type (Additional file 3: Table S1). The vast majority of DE *Bgh* genome mapped sRNAs and milRNA candidates (98.6 and 100%, respectively) were DE only at 48 HAI (Table 1). The *Bgh*-susceptible *mla6* mutant had significantly higher number of differentially expressed reads at 48 HAI than any other condition suggesting a large shift in sRNA regulation at that time point. However, the total number of *Bgh* genome mapped sRNAs was not significantly different among compatible and incompatible interactions at 48 HAI, suggesting that the peak of DE sRNAs at 48 HAI is unrelated to the relative biomass of *Bgh* (ANOVA with α = 0.05) (Additional file 1: Figure S1). At 48 HAI, compatible *Bgh* infections are transitioning to secondary hyphae production and potentially beginning the reproductive development program. The DE of sRNAs at this time point may represent a shift in gene expression towards reproductive capacity.

**Table 1:** Number of differentially-expressed, *Bgh* genome mapped sRNAs as compared to wildtype (CI 16151) at 0 to 48 h after inoculation

Genotype	Resistant or Susceptible	Time Point	Positive DE	Negative DE
*bln1-m19089*	Res	0	0	0
*mla6-m18982*	Sus	0	1	0
*rar3-m11526*	Sus	0	0	0
*(mla6 + bln1) m19028*	Sus	0	0	0
*bln1-m19089*	Res	16	0	1
*mla6-m18982*	Sus	16	0	17
*rar3-m11526*	Sus	16	0	5
*(mla6 + bln1) m19028*	Sus	16	0	2
*bln1-m19089*	Res	20	0	22
*mla6-m18982*	Sus	20	15	28
*rar3-m11526*	Sus	20	0	40
*(mla6 + bln1) m19028*	Sus	20	1	13
*bln1-m19089*	Res	24	0	2
*mla6-m18982*	Sus	24	2	4
*rar3-m11526*	Sus	24	0	0
*(mla6 + bln1) m19028*	Sus	24	0	2
*bln1-m19089*	Res	32	0	2
*mla6-m18982*	Sus	32	3	26
*rar3-m11526*	Sus	32	0	0
*(mla6 + bln1) m19028*	Sus	32	0	4
*bln1-m19089*	Res	48	0	4
*mla6-m18982* ^*a*^	Sus	48	8090	997
*rar3-m11526*	Sus	48	2433	285
*(mla6 + bln1) m19028*	Sus	48	1257	55

^a^Note that the *Bgh* infected barley line *mla6-m18982* at 48 HAI had significantly more DE sRNAs than all other genotype/time points tested (α < 0.001)

The majority (88.7%) of DE *Bgh* genome mapped sRNAs collected in time points before 48 HAI had negative differential expression. This may mean that the transcript targets of these sRNAs have higher active transcript counts relative to time points where these sRNAs are more highly expressed. The transcript targets of these sRNAs have not been predicted at this time, as they did not pass PARE validation filters.

### Bgh PARE-validated milRNAs and Bgh genome-mapped sRNA reads target genes in effector function and metabolic control

PARE is a high-throughput method for identifying in vivo sRNA cut sites [36]. The reads in PARE libraries represent a distribution of cleaved 3′ ends from poly-A-containing transcripts. Sequenced PARE libraries contained ~ 166 million raw reads that were filtered and mapped to the *Bgh* genome as described in Fig. 1b. The two programs sPARTA and CleaveLand were used to analyze the PARE sequencing data independently and identify high-confidence sRNA/transcript pairs [31, 32]. The output sRNA/transcript pairs were filtered using an adjusted *p*-value of less than 0.05 and a PARE category of less than 2 (reads were equal to the maximum for the target transcript). The *p*-value represents the likelihood that the miRNA is cleaved at that site, and, based on complementarity, the read abundance at the exact site where the miRNA is predicted to target, as well as the background of off-target reads adjacent to this cleavage site. The results of the specified filters include a total of 230 pairs (192 PARE-validated milRNAs and 149 unique *Bgh* transcripts) with high likelihood of interaction resulting in transcript cleavage (summarized in Additional file 4: Table S2). Functional annotation of the target transcripts was accomplished using available Ensembl annotations, BLASTX searches, Interproscan annotation (version 5.15–54-0), and literature review (Additional file 5: Table S3). Annotations of *Bgh* PARE-validated sRNA targets are shown in Table 2 and include effectors (19.5%), metabolism (14.8%), translation-related (12.1%), and signaling (7.4%) as contrasted with the most prevalent barley targets denoted as transcriptional regulation (33.3%), unknown (13.8%), signaling (11.4%), and metabolism (8.1%).

**Table 2:** Functional annotation of PARE-validated *Bgh* and barley sRNA transcript targets

Functional Category	*Bgh* Count	*Bgh* %	Barley Count	Barley %
Effector	29	19.5	0	0
Metabolism	22	14.8	10	8.1
Hypothetical/Unknown	20	13.4	17	13.8
Translation-related	18	12.1	3	2.4
Signaling	11	7.4	14	11.4
Transporter	10	6.7	5	4.1
Cellular Structure/Function	9	6	8	6.5
Transcriptional Regulation	6	4	41	33.3
Protein Folding	6	4	0	0
Vesicle Transport	5	3.4	3	2.4
Protein Turnover	4	2.7	1	0.8
Energy-related	4	2.7	8	6.5
Post-Translational Modification	3	2	1	0.8
Redox Control	2	1.3	2	1.6
Defense	0	0	5	4.1
Cell Wall-Related	0	0	5	4.1
Total	149	100	123	100

The effector category contains ten CSEP members and twelve members of the EKA family. Several of the predicted CSEP targets, including CSEP0008 (*AVR*_*a1*_) and CSEP0196 (BEC1040), have published functions in *Bgh* pathology [37, 38]. Several of the DE milRNAs are predicted to regulate effector genes and are upregulated at 48 HAI. This may be related to a change in effector expression associated with a transition in lifestyle from primary infection to secondary hyphal growth and reproduction. Homologs of many CSEP and EKA effectors are only found in powdery mildews, and many are undergoing positive selection pressure [5, 10]. These properties indicate that they are both important to powdery mildew biology and subject to rapid evolution. In *Phytophthora sojae* the avirulence factor Avr3a is silenced by sRNAs, leading to infection of plants carrying the *R*-gene *Rps3a* [25]. In a similar manner, the silencing of effector genes may allow selective escape of barley resistance factors.

Metabolic targets were spread across many facets of primary metabolism, such as amino acids, fatty acids, carbohydrates, and nucleic acids. This broad cross-section of metabolic gene targets indicates that *Bgh* may be controlling long-term metabolic flow with sRNAs in a similar fashion as plants and animals [39, 40]. In one example of metabolic control, a transcript encoding a NAD(+)-dependent glutamate synthase is predicted to be cleaved in one location by seven different sRNAs located at independent loci in the *Bgh* genome. Control of nitrogen metabolism is especially important as *Bgh* lacks enzymes related to the assimilation of nitrate [8]. The translation-related category comprises many members that are either components of ribosomes or regulation of translation. Control of translation components would allow active gene expression of infection related transcripts without the metabolic cost associated with protein production until they are needed in the infection process. Members of the signaling category include several kinases and calcium signaling-related proteins. Calcium signaling has been shown to be important for successful infection in plant fungal pathogens such as *Magnaporthe oryzae* [41].

### Regulation of Bgh EKA family members through embedded PARE-validated hairpin RNA

**Fig. 2:**
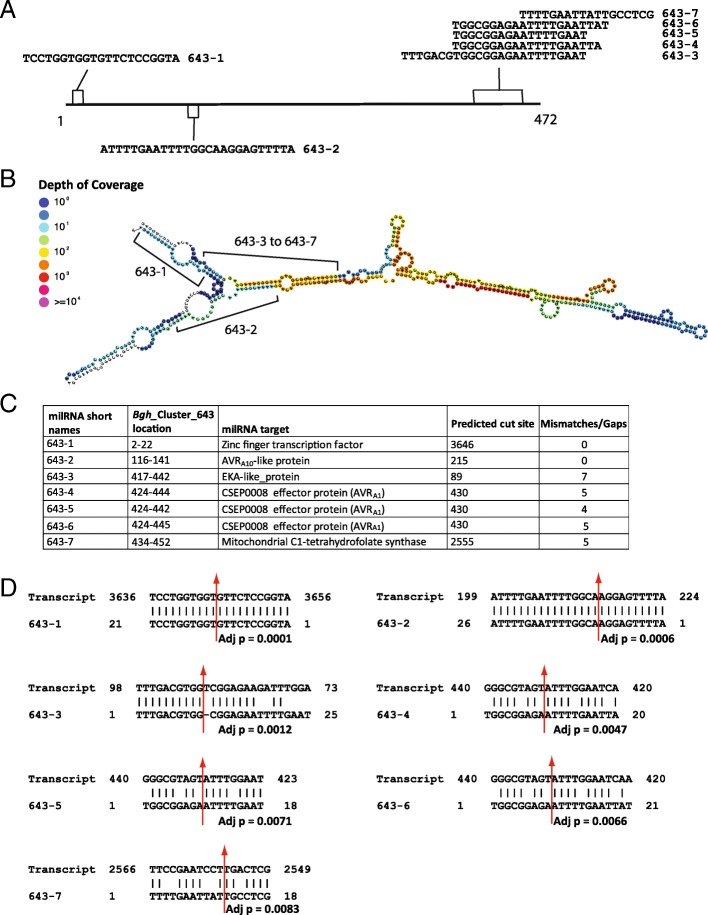
*Bgh*_Cluster_643 structure and encoded PARE-validated milRNAs. **a** Linear representation of *Bgh*_Cluster_643 with milRNA encoding regions for 643–1 to 643–7 highlighted. **b** RNAfold predicted *Bgh*_Cluster_643 structure with sRNA mapping density scale from blue (no coverage) to purple (> = 10^4^ mapping reads) outputted from the ShortStack [34]. **c** Details of *Bgh*_Cluster_643 predicted milRNAs including name, location on *Bgh*_Cluster_643, predicted transcript target annotation, and number of mismatches/gaps in transcript alignment. Note that in Additional file 4: Table 2, Column “A”; lines 195–206 show the original designations from the ShortStack program, while simplified names used here are shown in parentheses. **d** Alignments of predicted milRNA to their transcript targets / cleavage sites with adjusted *p* values (detailed in Additional file 4: Table 2). Cleavage sites are represented by red arrows

**Fig. 3:**
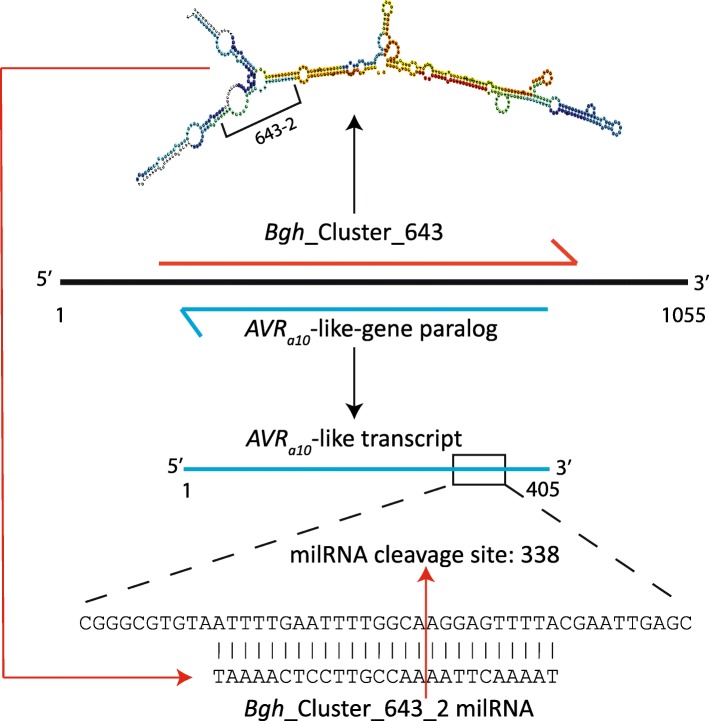
*Bgh* genome supercontig HF944340 encodes both a predicted natural antisense siRNA (natsiRNA) transcript as well as a member of the EKA effector gene family. The *Bgh*_Cluster_643 natsiRNA transcript is processed into several milRNAs candidates including *Bgh*_Cluster_643–6. The EKA transcript (BGHDH14_bgh06737) is encoded antiparallel to the hairpin and is transcribed and targeted for transcript cleavage by *Bgh*_Cluster_643–2

A hairpin forming precursor designated *Bgh*_Cluster_643, identified with the ShortStack program, encodes seven PARE-validated milRNAs that are predicted to target seven different *Bgh* transcripts (Fig. 2). Three of these predicted targets encode effectors including two EKA family members, as well as the candidate secreted effector gene *CSEP0008*. *CSEP0008* encodes the avirulence protein AVR_A1_ that is recognized by the R-protein MLA1 [37]. One of the other *Bgh*_Cluster_643 encoded sRNA targets is the *AVR*_*a10*_-like gene (BGHDH14_bgh06737). The AVR_A10_-like protein is a member of the EKA effector family and has 861 homologs in the *Bgh* genome at a BLASTn e-value cut-off of 1e-100. The EKA effector family open reading frames are located within an active LINE-type TE, and are spread across the *Bgh* genome [5]. Some EKA family members actively encode transcripts, but many are inactive. We identified 20 homologs of the *AVR*_*a10*_-like gene (BGHDH14_bgh06737) that are encoded in genomic loci overlapping with a homolog of the hairpin precursor *Bgh*_Cluster_643 (BLASTn e-value cut-off of 1e-100) on the opposite strand (Additional file 6: Table S4). Each of these overlapping sequences have exact matching reverse complementary portions with non-overlapping overhangs**.** The length of these overlaps, and the hairpin nature of the *Bgh*_Cluster_643 homologs suggests a mechanism for control of these EKA family members in a manner similar to natural antisense miRNAs (nat-miRNAs) in plants [42]. The proposed model for regulation of EKA family members through opposite-strand encoded hairpin RNA is shown for *Bgh*_Cluster_643 and an *AVR*_*a10*_-like gene in Fig. 3**.**

### Bgh differential genic vs non-genic sRNA mapping

**Fig. 4:**
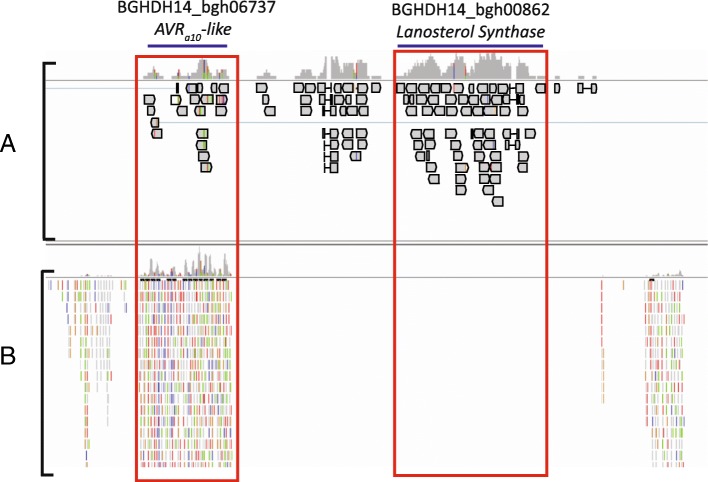
Transcript and sRNA sequencing reads mapped to *Bgh* genome positions near BGHDH14_bgh06737 and BGHDH14_bgh00862. The gene transcript models are highlighted with the blue lines, while the transcript and sRNA reads for each gene are highlighted with the red boxes. **a** Transcript based RNA-Seq reads mapped to the *Bgh* genome. **b** sRNA based RNA-Seq reads mapped to the *Bgh* genome

We explored the mapping frequency of *Bgh* genome mapped sRNAs both inside and outside of predicted gene models. The supercontigs from the ensembl *Bgh* genome (v32) were divided into genic and non-genic portions, based on the predicted gene models, resulting in 6469 predicted gene segments, and 13311 non-genic segments. The average mapping sRNA density was 15.6 read/Kb for genic segments and 1767.6 for non-genic segments. In fact, 84.6% of all predicted gene models had no mapped reads, as compared with 14.1% in non-genic segments. In many cases there are regions of high sRNA mapping upstream and downstream of predicted transcripts. There are exceptions to this general trend, as demonstrated by the *AVR*_*a10*_-like gene (BGHDH14_bgh06737) and the 20 homologs with predicted overlapping hairpins. These potential EKA family members have a predicted mapping density of 4702.7 read/Kb, which can be explained by the presence of the hairpin sequences located on the opposite strand to the EKA gene homologs. As an example, Fig. 4 illustrates the RNA-Seq transcripts, along with sRNA-Seq mapping data for *AVR*_*a10*_-like gene (BGHDH14_bgh06737) and its immediate downstream lanosterol synthase gene (BGHDH14_bgh00862). The lanosterol synthase gene has zero mapped sRNA-Seq reads, while the *AVR*_*a10*_-like gene has over 4300 mapped sRNA-Seq reads. The functional significance of the sRNA mapping frequencies inside and outside of genic regions is unclear at this time, but one possible explanation is active silencing mechanisms functioning on transposable elements that surround areas of active transcription.

### Differential regulation of reactive oxygen species-related barley miRNAs

Differential expression (DE) of barley predicted miRNAs or barley genome mapped sRNAs at each time point were identified by comparing WT CI 16151 to the four mutant lines using the DESeq2 program [35]. The DESeq2 outputs were filtered for adjusted *p*-values of less than 0.05. Out of 1425 predicted barley miRNAs, there are 730 unique sequences. Of these sequences, 9 (1.2%) are DE during at least one time point (Table 3). Out of the 9 unique sequences, 4 have homology to miRNA families including miR2120, miR398, and miR528. Both miR398 and miR528 have been linked to control of the reactive oxygen species (ROS) related genes *chloroplast copper/zinc superoxide dismutase 1* (*HvSOD1*) in barley and *L-ascorbate oxidase* (*AO*) in rice [43, 44]. The miRNA target site of rice *AO* (XM_015787755.1) from Wu et al. (2017) is located in the 3′ UTR, and is not conserved in any barley *AO*, so it is unclear if overexpression of barley miR528 in the *mla6* mutant is related to ROS regulation. However, several other studies have indicated that miR528 is involved in regulation of ROS through a copper super oxide dismutase gene and other targets [45, 46].

**Table 3:** Differentially expressed predicted miRNAs and barley mapped reads with homology to miRBase miRNAs

Predicted miRNA or read	Sequence	miRBase match	Number of predicted barley copies	DE time points (and log_2_ fold changes)	miRBase blastn overlap	Mis-matches
DE barley mapped read	TCGGACCAGGCTTCATGCCCC	miR165	NA	*bln1* 16 HAI (-2.55) , *bln1* 20 HAI (-2.39), *mla6* 20 HAI (-1.77), *rar3* 20 HAI (-2.14), *bln1* 24 HAI (-2.47), *mla6* 24 HAI (-2.02), *bln1* 48 HAI (-2.41), *mla6* 48 HAI (-1.78)		1
DE barley mapped read	TTCGGACCAGGCTTCCTTCCC	miR166	NA	*mla6* 48 HAI (1.92)		2
DE barley mapped read	TGGGACCAGGCTTCATTCCCC	miR166	NA	*bln1* 20 HAI (-2.24), *rar3* 20 HAI (-1.71)		1
DE barley mapped read	TCGGACCAGGGTTCATTCCCC	miR166	NA	*bln1* 48 HAI (-2.31), *mla6* 48 HAI (-1.80)		1
DE barley mapped read	TTCGGACCAGGCTTCAGTCCC	miR166	NA	*rar3* 48 HAI (-2.10)		2
DE predicted miRNA	ACACAAACCGGGACTAAAG	miR2120	9	*mla6* 20 HAI (1.59)		2
DE predicted miRNA	GTGTTCTCAGGTCGCCCCCGC	miR398	2	*mla6* 32 HAI (2.03)		1
DE predicted miRNA	AGAACAGAGAATGGCGATAGACTC	miR398	1	*mla6* 0 HAI (1.63), *mla6* 20 HAI (1.72), *mla6* 24 HAI (1.66), *mla6* 48 HAI (1.93)		4
DE barley mapped read	TGTGTTCTCAGGTCGCCCCCG	miR398	NA	*mla6* 24 HAI (1.71), *mla6* 32 HAI (2.57)		0
DE predicted miRNA	TCCTGTGCCTGCCTCTTCCAT	miR528	1	*mla6* 20 HAI (1.97), *mla6* 24 HAI (2.27), *mla6* 32 HAI (2.18)		1
DE barley mapped read	TCCTGTGCCTGCCTCTTCCAT	miR528	NA	*mla6* 24 HAI (2.07), *mla6* 32 HAI (2.19)		1
DE barley mapped read	TGGAAGGGGCATGCAGAGGA	miR528	NA	*mla6* 32 HAI (1.86)		0
DE barley mapped read	TGGAAGGGGCATGCAGAGGAG	miR528	NA	*mla6* 16 HAI (2.20), *mla6* 20 HAI (2.40), *mla6* 24 HAI (2.21), *mla6* 32 HAI (2.09)		0
DE barley mapped read	CCTGTGCCTGCCTCTTCCATT	miR528	NA	*mla6* 0 HAI (1.99)		0
DE predicted miRNA	ATTTTGCTTCGTATGTAGACT	none	17	*mla6* 0 HAI (1.97)	none	NA
DE predicted miRNA	TATTAGTTGACAGAGGGAGTA	none	5	*mla6* 48 HAI (-1.77), *mla6-bln1* 48 HAI (-2.44), *bln1* 48 HAI (-2.40)	none	NA
DE predicted miRNA	AACTAGTACTACTCTAATGTGCCT	none	3	*mla6* 0 HAI (-1.07)	none	NA
DE predicted miRNA	GCTTTCATAGCTCAGTTGGTTAGAGCACCCG	none	1	*bln1* 32 HAI (1.64)	none	NA
DE predicted miRNA	AATTTGAACTGTGAAACT	none	1	*mla6* 0 HAI (1.46), *mla6* 20 HAI (1.76), *mla6* 24 HAI (1.56)	none	NA

Out of 1,980,623 unique barley mapped sRNAs, 2423 were differentially accumulated in at least one time point (Additional file 7: Table S5). These include 13 reads that have homology to three conserved miRNA families including miR165/miR166, miR398, and miR528, (Table 3). Members of the miR165/miR166 family regulate a HD-ZIPIII transcription factor important for plant development, and have been shown to be positively regulated during pathogen infection [47]. In barley, the MLA6 R-protein regulates the expression of miR398, which in turn, controls ROS levels through differential expression of *chloroplast copper/zinc superoxide dismutase 1* (*HvSOD1*) [44]. Down-regulation of ROS responses controlled by miR398 and miR528 in the susceptible *mla6* mutant would allow for more favorable infection conditions for *Bgh*.

### Barley PARE-validated sRNA cleavage of transcription factors and signaling-related transcripts

The PARE analysis programs utilize barley transcriptome data, candidate sRNAs, and quality-trimmed PARE sequencing data to identify validated sRNA-transcript pairs. Through this process we identified three types of PARE-validated sRNAs (Additional file 8: Table S6). First, we identified 24 conserved miRNAs with known transcript targets. Second, we identified 35 novel miRNAs with PARE-validated cut sites. Lastly, we identified 61 barley mapping DE reads with PARE-validated cut sites. The transcript targets for the PARE-validated sRNAs were functionally annotated using Ensembl annotations, blastx comparisons to the nr database, interproscan (v 5.15–54-0), and literature review (Table 1). Transcriptional regulation, signaling, and energy-related functional categories made up 33.3, 11.4, and 6.5% of the functional annotations, respectively. Transcription-related targets included development-related transcription factors (TFs), auxin response factors, homeobox, MYB, and NAC TFs, as well as transcript splicing factors. sRNAs targeting signaling functions included calcium, phosphate (kinases and phosphatases), and phytohormones including JA and auxin. In the energy-related category, photosynthesis related genes are targeted including three isoforms of cytochrome f, four oxidoreductases, and a component of the photosystem antenna complex. Many of these transcriptional regulators, signaling components, and photosynthesis genes may be co-regulated during infection to control growth rates, as defense responses require relatively large energy investments [48].

### Barley leaf phased siRNAs are predicted to regulate gene expression

Phased siRNAs (phasiRNAs) in plants are commonly 21 or 24 nucleotide (nt) sRNAs derived from both coding and non-coding transcripts. Monocots primarily produce phasiRNAs in reproductive tissues that regulate non-coding RNA expression [18, 49]. However, very few studies have reported regulation of gene expression in non-*TAS* loci in monocots with some exceptions [24, 50]. In our study of *Bgh*-infected barley leaves we identified barley phasiRNA loci with phasing sizes of mostly 24 nt that overlap with protein coding transcripts with functional categories including metabolism and defense-related signaling.

**Fig. 5:**
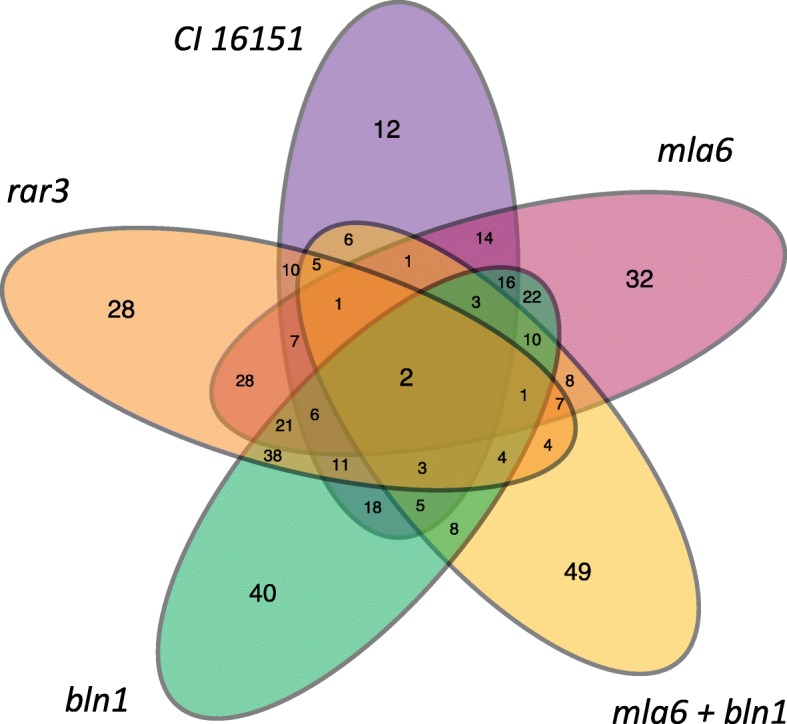
Genotype membership distribution for genotype-specific phasiRNA loci. CI 16151 is designated by purple, *mla6* by pink, *rar3* by orange, *bln1* by green, and *mla6 + bln1* by yellow

To identify barley phasiRNA loci expressed under *Bgh* infection, we mapped sequencing reads from all 90 Illumina sRNA libraries to the barley genome with no mismatches allowed using the bowtie program [51]. These mapped reads were run through two filters described in [52] and detailed in Methods. First, the *p*-value filter was applied to identify loci with a *p*-value of < 0.001. Second, a phasing score was calculated for a 1 Kb region surrounding these loci. These filters were used to identify phasing sites at the genotype level. We identified 1274 individual phasiRNA loci with a high frequency (88.9%) of 24 nt phasing size (Additional file 2: Figure S2). Physically overlapping phasiRNAs were concatenated to form 420 total phasing loci. The mapped locations of the concatenated phasiRNA loci were compared to predicted barley protein-encoding genes, miRNA genes, ncRNA-encoding loci, and transposable elements. The concatenated phasing loci did not overlap with miRNA loci from this study, the barley genome described in Mascher et al. 2017, nor with barley ncRNAs from Ensembl (v39). However, we did uncover 48 out of 420 phasing loci (11.4%) that had overlaps with predicted barley TEs [53]. We also found that 225 of the 420 phasiRNA loci (53.6%) overlapped within 1 Kb of 220 barley transcripts. Out of the 420 phasiRNA loci, 161 (38.3%) are uniquely expressed in one genotype pool, while 259 loci (61.7%) were expressed in at least two conditions (Fig. 5). The protein coding transcripts with phasiRNA loci overlapping them had a mix of functional categories including signaling, metabolism, transcription-related, and cellular structure and function (Table 4).

**Table 4:** Barley phasiRNA transcript target annotations

Functional Category	Number	Percentage
Signaling	41	18.7
Metabolism	37	16.9
Hypothetical or unknown	36	16.4
Transcription-related	24	11.0
Cellular structure and function	20	9.1
Defense	12	5.5
Protein turnover	12	5.5
Vesicle transport	9	4.1
Energy-related	7	3.2
Transporter	7	3.2
Cell wall-related	4	1.8
Redox control	4	1.8
Protein folding	2	0.9
Translation-related	2	0.9
Post translational modification	1	0.5
Stress related	1	0.5
Total	219	100

Eight NLRs and 24 receptor-like kinases are overlapped by phasiRNA loci. Fisher’s exact test was applied to show the proportion of receptor-like kinases overlapped by phasiRNA loci is significantly enriched (10.9%) compared with the total receptor-like kinases barley genome (2.6%). This comparison was carried out based on the proportion of Ensembl annotations from predicted phasiRNA transcript overlaps compared to the proportion of total Ensembl annotated barley transcripts that had receptor-like kinase annotations. This suggests that phasiRNA regulation of receptor-like kinases during *Bgh* infection may be an important regulatory feature.

**Fig. 6:**
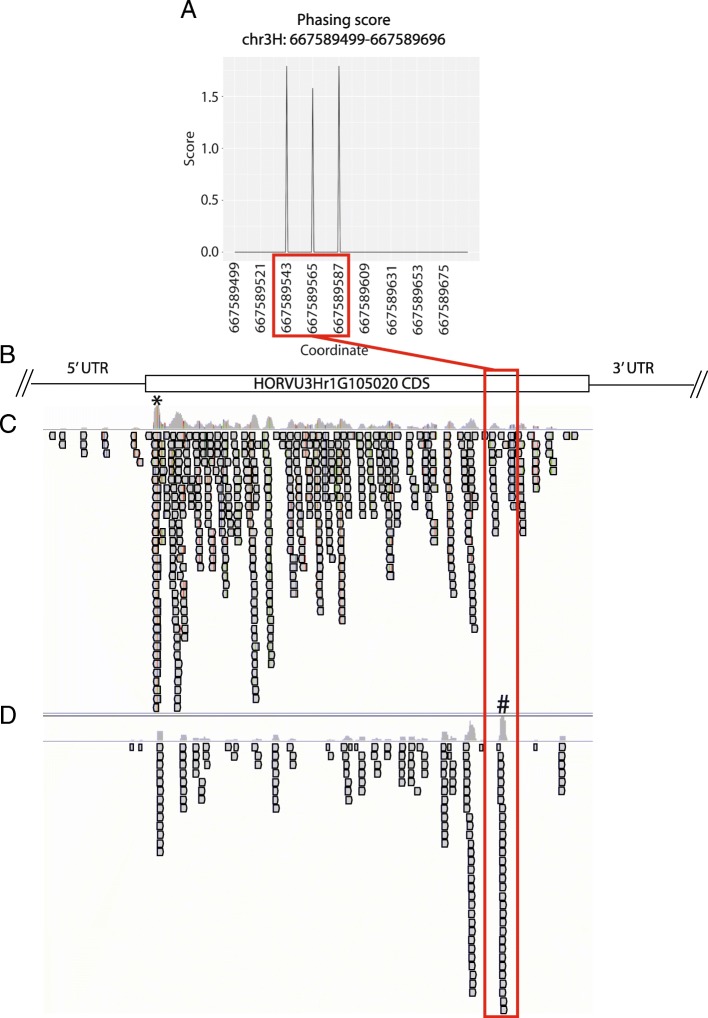
PhasiRNA locus phasing score and mapping position relative to barley gene HORVU3Hr1G105020, a NLR gene with homology to wheat *CNL9*. **a** Phasing score diagram on chromosome 3 from 667589499 to 667589696. **b** Gene model section of HORVU3Hr1G105020 overlapped by phasiRNA loci. **c** sRNA data from panel mapped to barley genome. Maximum sRNA mapping depth of 33 reads at peak highlighted with a * **d** PARE library data from panel mapped to barley genome. The phasiRNA seed region is highlighted with the red boxes. Maximum PARE read mapping depth of 49 reads at peak highlighted with #

One example of the NLR transcripts overlapped by phasiRNA loci, HORVU3Hr1G105020, is of special interest because of its high level of amino acid identity (84%) with *CNL9* from wheat. *CNL9* encodes the CC-NLR protein responsible for *SR35* resistance to Ug99 wheat stem rust [54]. The barley gene HORVU3Hr1G105020 is one of two potential barley NLRs with a blastx e-value match to *CNL9* of greater than 1e-100. The location of a predicted phasiRNA locus overlapping HORVU3Hr1G105020 coincides with substantial sRNA accumulation in the coding region of the NLR-encoding transcript (Fig. 6).

## Discussion

### Small RNA profiling of Bgh infected barley leaves

Gene expression during pathogen infections can change dramatically for both the host plant and the invading pathogen. These changes can come in several forms including alteration of carbon flow and other metabolic processes, altered transcription factor profiles, and changes in the levels of defense or virulence related genes. These processes can be dramatically regulated by sRNAs including both siRNA and milRNA/miRNA types.

In this study we sought to understand how sRNAs in barley and *Bgh* affect gene expression during infection in both the pathogen and the host. To address this question we compared sRNA profiles of both barley and *Bgh* isolate 5874 across five barley lines from a total of 90 sRNA sequencing libraries. Two independent approaches were taken to identify potentially biologically important sRNAs. First, plant rules-based miRNA prediction programs were used to predict barley and *Bgh* candidate milRNAs/miRNAs and second, reads were identified that mapped exactly to the barley or *Bgh* genome, had at least ten counts across all libraries, and were DE in at least one line compared to wild type during at least one time point. These two approaches yielded 1741 *Bgh* milRNA candidates and 13,311 DE *Bgh* genome mapped sRNAs along with 1,425 barley miRNA candidates and 2,423 DE barley genome mapped sRNAs. To complement the sRNA sequencing data, we employed the parallel analysis of RNA ends (PARE) to authenticate predicted transcript cleavage sites in vivo for both the milRNA candidates and the DE *Bgh* genome mapped sRNAs [36]. PARE data was used to identify 230 likely pairs of *Bgh* sRNAs and *Bgh* transcripts along with 120 likely pairs of barley sRNAs and barley transcripts.

### Bgh small RNA differential accumulation at 48 HAI

Of the 268 DE milRNA candidates and 13311 DE *Bgh* genome mapped sRNAs, we found that 100 and 98.6%, respectively, were only DE at 48 HAI, and only in compatible interactions (Table 1). This finding is curious, given that we have identified significantly DE transcripts at every time point. This could be interpreted that the wave of DE in *Bgh* sRNAs at 48 HAI is related to a developmental transition in successful infections (*i.e*., compatible interactions). This may be an important transition point in the infections, where *Bgh* is moving from nutrient acquisition and defense suppression towards secondary hyphal growth, reproduction, and a new wave of effector expression. This developmental stage change may require a different set of proteins for proper growth, and therefore a specific set of sRNAs is significantly upregulated to quickly reduce target transcript levels.

### Bgh sRNAs are predicted to control effector and metabolism-related gene expression

Through a combination of the sRNA sequencing and PARE data, we identified several highly enriched target annotations related to successful barley infection, including effectors and metabolic genes (Table 2). Fungal effector proteins in plant pathogens are vital for both reducing defense responses and nutrient acquisition. *Bgh* has two effector types, CSEPs and EKAs, that have 722 and ~ 1350 copies each [10, 55]. The combination of these potential effector genes represent ~ 30% of the predicted genes overall for *Bgh*. *Bgh* effectors are especially important for successful infection of barley, as reducing expression of even a single effector can significantly affect pathogenicity [56–58]. About 20% of all PARE-validated targets in our filtered set were effectors. These potential targets include AVR_A1_, the cognate avirulence effector to barley MLA1 [37, 59]; *CSEP0196 (BEC1040)*, an effector that when knocked down with host induced gene silencing (HIGS) results in significant reduction in *Bgh* pathogenicity [38]; several additional CSEPs, and a dozen members of the EKA effector family. Differential regulation of these particular CSEP and EKA encoding genes at 48 HAI and after may be important in the transition from survival to reproduction.

Control of metabolism through miRNAs has been shown extensively in plants and animals. Throughout the developmental cycle of *Bgh*, timed expression of metabolic genes is important for both survival and successful infection of barley. Key enzymes in fatty acid, nucleic acid, and amino acid biosynthesis along with nitrogen assimilation and carbon metabolism are potentially controlled through PARE-validated milRNAs. Silencing gene expression post-transcriptionally through sRNAs may allow for rapid regulatory changes that immediately reduce protein biosynthesis levels, as opposed to transcriptional gene silencing. One important example for metabolic control is glutamate synthase, a key enzyme in nitrogen assimilation. Glutamate synthase is especially important in *Bgh* as many of the other enzymes in nitrogen assimilation are have been lost over evolutionary time [8]. The glutamate synthase enzyme was recently shown to be important in *Magnaporthe oryzae* pathogenesis of rice [60]. In *M. oryzae* glutamate synthase knockouts, both appressorial penetration as well as hyphal spread were significantly reduced. In our study we identified seven separate PARE-validated milRNAs that cleave glutamate synthase transcripts. The nitrogen status of *Bgh* can vary greatly, depending on its infection status of barley. These milRNAs may allow *Bgh* to control the flow of nitrogen depending on its availability.

### A subset of the Bgh EKA effector family is potentially controlled by sRNAs

The milRNA encoding hairpin *Bgh*_Cluster_643 is biologically significant for three reasons. First, hairpin *Bgh*_Cluster_643 encodes seven milRNA candidates that are predicted to target eight different *Bgh* genes for cleavage, including three effector proteins. Second, *Bgh*_Cluster_643 is encoded in an antiparallel orientation to one of its encoded milRNA predicted targets: *AVR*_*a10*_-like gene (*BGHDH14_bgh06737*). We have identified 20 additional EKA family members that are highly similar to the *BGHDH14_bgh06737* gene that also encode hairpins highly similar to *Bgh*_Cluster_643. These hairpin-forming EKA family members may be functioning through a mechanism similar to that proposed to natsiRNAs in plants [61]. Other TE-related gene families may reveal similar examples. And third, the 20 genomic positions have significantly higher sRNA mapping density than other predicted genic positions in the genome. We found that the 20 hairpin positions have an average density of 4702.7 reads/Kb, compared with the average genic positions of 15.6 read/Kb. This suggests that these positions are highly regulated by sRNAs.

### Barley PhasiRNA loci are correlated with diverse pathways including receptor kinases and transcription factors

PhasiRNAs are secondary siRNAs that can silence transcripts in both *cis* and *trans*. They are produced when a RISC-bound miRNA targets a transcript leading to the production of double stranded RNA by RDRP, and cleavage by DCL into phased siRNAs. Most phasiRNA loci in grasses are associated with silencing ncRNA in reproductive tissues [49]. Two notable exceptions include the *TAS3* tasiRNA locus that regulates auxin response factors and the barley *Mla* resistance gene [23, 24]. The identified phasiRNA loci in *Bgh* infected barley leaves overlap substantially with protein coding transcripts, but do not overlap with known ncRNAs or miRNAs in barley, and very little overlap with TEs. Although there are phasiRNA overlaps with *NLR* defense genes, the low numbers suggest a lack of a general *NLR* phasing mechanism as compared with eudicots [62]. A high number of receptor-like kinase gene targets suggests a different mechanism for defense gene regulation in barley.

We found that 225 of the 420 predicted phasiRNA loci (53.6%) were located within 1 Kb of predicted barley protein-encoding transcripts. Of these phasing loci, 161 (38.3%) were only found in single barley genotypes [Fig. 5; CI 16151 (12/420 = 2.9%), *mla6* (32/420 = 7.6%), *rar3* (28/420 = 6.7%), *bln1* (40/420 = 9.5%), *mla6 + bln1* (49/420 = 11.7%)]. This relatively high proportion of genotype-specific phasing loci suggests that responses to *Bgh* infection can be genotype specific, with each barley mutant responding differently according to the immune signaling pathway that has been affected. It has previously been demonstrated that different alleles of *Mla*, in addition to other genes involved in disease resistance signaling, have a profound effect on downstream gene expression when challenged with the same *Bgh* 5874 isolate [29, 63]. Thus, it is not surprising that this variation would extend to the phasing loci seen in this study.

Functional categories highly represented in the data include signaling, metabolism, and transcription-related at 19, 17, and 16%, respectively. In the signaling category receptor-like kinases are significantly over-represented in the genotype-specific phasiRNA targets when compared with the current barley annotated transcriptome, which may indicate a novel mechanism of pathogen defense regulated by phasiRNAs in barley. Several barley receptor-like kinase genes are involved in pathogen response including *Reaction to Puccinia graminis 1* (*Rpg1)*, *rat sarcoma homolog binding protein kinase* (*RBK1)*, *somatic embryogenesis receptor-like kinase 2* (*SERK2)*, *LRR/malectin receptor-like kinase* (*LEMK1)*, and *cysteine-rich receptor-like protein kinase 1* (*CRK1*) [64–68]. In dicots, *NLR* defense genes are regulated by phasiRNAs triggered by miRNAs targeting conserved portions of *NLR* transcripts [62]. For example in a recent study on soybean sRNAs, the authors found 41% of *PHAS* loci overlapped with *NLR* genes [69]. In our genotype-specific phasing data, we found only 4% of the phasiRNA loci overlapped with *NLR* genes. Our data shows that barley leaves infected with *Bgh* produce phasiRNA potentially regulating a diverse set of genes affecting metabolism, transcription, signaling, and defense. Previous studies on phasiRNAs in grasses (besides *TAS3*) have focused almost exclusively on phasiRNAs targeting ncRNAs in reproductive tissues [70–73]. The results in this report indicate that barley phasiRNAs overlap extensively with protein-coding transcripts, and that defense response genes including receptor-like kinases are potentially regulated by phasiRNAs.

### PARE-validated sRNAs targeting transcription, signaling, and photosynthesis

We produced PARE libraries from genotype-pooled *Bgh* infected barley leaf RNA to confirm predicted sRNA transcript cut sites in vivo. We identified 24 PARE-validated miRNAs, representing eight conserved miRNA families including miR156, miR159, miR160, miR164, hvu-miR165/hvu-miR166, miR169, miR171, and miR396. We further identified 35 novel barley miRNAs and 64 DE barley genome mapped sRNAs with PARE-validated cut sites (Additional file 8: Table S6). The majority of conserved plant miRNAs target transcription factors [74, 75], which matches well with our data. The eight conserved miRNA families identified in the PARE data all target transcription factors with roles in development and biotic stress responses [76–86]. The transcription-related genes regulated by the PARE-validated sRNAs encode several families of transcription factors including Homeobox, MYB, NAC, ARF, GRAS, bZIP, squamosa promoter-binding-like, and factors related to transcript splicing. These results indicate that transcription factor encoding genes are being cleaved during *Bgh* infection. However, significant differences in accumulation were not found for the miRNAs targeting these gene transcripts in our data. This may mean that the changes in transcription-factor genes in the *mla6*, *rar3*, *bln1,* and *mla6* + *bln1* mutant lines are largely not due to differences in expression of regulatory miRNAs.

Additionally, signaling and energy categories were highly represented as regulatory targets of the PARE-validated sRNAs. The signaling transcript targets included proteins involved in phosphate signaling (kinases, receptor-like kinases, and phosphatases), calcium signaling (calmodulin and calcineurin B), and hormone signaling (JA and auxin). Hormone levels are changed as part of the PTI defense response to pathogen challenges in plants [87]. For example JA and Auxin function can be downregulated during infection by biotrophic pathogens to reduce growth rates, and promote the effects of SA [88]. The members of the energy-related category all are directly involved in photosynthesis, including members of the cytochrome f family, NADH-plastoquinone oxidoreductases, and the CP43 chlorophyll apoprotein. In response to pathogen infections, transcripts encoding photosynthetic machinery are generally downregulated [89]. However, photosystem proteins generally are very stable, which allows an infected plant to divert resources to defense, while maintaining active photosynthesis [90].

### Differentially accumulated sRNAs regulate PTI-related redox responses

Analysis of predicted miRNAs and barley genome mapped sRNAs identified several conserved miRNA families regulated during *Bgh* infection including miR166/165, miR398, and miR528. The miR166/165 family has diverse roles in development and response to stress through regulation of the Class III homeodomain-leucine zipper (HD-ZIP III) encoding transcripts [91, 92]. Multiple members of the highly conserved miR166/165 family are present in the genome of many plant species with diverse expression patterns [93–96]. The differential accumulation of members of the miR166/165 family have been associated with multiple stress responses including drought, cold, and pathogen challenge [47, 97–99]. We identified 5 different barley genome mapped sRNAs with homology to members of the miR166/165 family that were differentially expressed in at least one time point and barley immune signaling mutant compared with the wild-type progenitor. Four out of five barley genome mapped sRNAs had significant decreases in accumulation relative to wildtype in at least one condition, while the sixth had a significant increase (Table 3). In a recent study miR166/165 family member-specific was studied and it was discovered that some family members are strongly upregulated in susceptible lines, whereas others are downregulated [98]. It is unclear at this time what role downregulation of miR166/165 means for the CI 16151-derived mutants in our study, as both resistant (*bln1*) and susceptible lines (*mla6* and *rar3*) have significant downregulation relative to wildtype in at least one time point.

miR398 targets two copper superoxide dismutase gene transcripts as well as a cytochrome c oxidase [44, 100]. The regulation of miR398 has been shown to be important in stress responses including heat, drought, high salt, ABA, and pathogen challenge, amongst others [101]. In barley hvu-miR398 targets the *HvSOD1* transcript and is regulated by both *Mla* and *Rom1* in response to *Bgh* infection [44]. In our study, two predicted miRNAs and one barley genome mapped sRNAs with homology to miR398 were significantly upregulated in the line carrying the *mla6* mutation. These data support the findings of Xu et al. (2014) in that miR398 is upregulated in the *mla6* mutant as compared with the wild-type progenitor **(**Table 3), leading to a suppression of *HvSOD1*.

The miRNA miR528 has been experimentally shown to target transcripts encoding L-ascorbate oxidase in rice [43], plastocyanin-like blue copper ion binding protein in sugarcane [102], and the F-box/LRR-repeat protein MAX2 in rice [103]. miR528 has been associated with embryo development, metal toxicity, oxidative stress, drought stress, salt stress, and pathogen challenge [43, 45, 104–108]. Similar to miR398, we found one predicted miRNA and four barley genome mapped sRNAs with homology to miR528 to have significantly increased accumulation in the *Bgh* susceptible *mla6* mutant. The role of miR528 in Poaceae pathogen defense appears diverse as it was upregulated in both resistant and susceptible wheat lines challenged with leaf rust and powdery mildew [109, 110]. In our study, however, the accumulation of miR528 is significantly increased in the susceptible *mla6* barley mutant. This upregulation of miR528 in *mla6* could contribute to a reduced ROS response to *Bgh* infection, similar to that described for miR398 [44].

## Conclusions

In this study we sought to identify sRNAs that are involved in the regulation of gene expression during *Bgh* infection of barley leaves. To complete its lifecycle *Bgh* has to suppress barley defenses while taking up nutrients. When confronted with a *Bgh* infection barley epidermal cells reprogram their metabolism and activate defense processes. Data in this report supports that many of these processes in barley and *Bgh* are regulated by sRNAs. We identified sRNAs in both species that are predicted to target genes involved in metabolic processes as well as defense/virulence proteins. These findings will contribute to our understanding of the complex interactions between obligate biotrophs and plant hosts.

## Methods

### Fungal and plant material

The CI 16151 barley line was created by introgression of the *Mla6* allele into universal susceptible cv Manchuria [111] and is resistant to *Blumeria graminis* f. sp. *hordei* (*Bgh*) isolate 5874 (*AVR*_*a1*_, *AVR*_*a6*_, *vir*_*a8*_, *AVR*_*a12*_, *vir*_*a13*_, *AVR*_*La*_). Mutant derivatives of CI 16151 were created through fast-neutron mutagenesis as described previously [28]. *Mla6* recognizes the AVR_A6_ effector from *Bgh* isolate 5874; plants with this allele are resistant and *mla6* mutants are susceptible [28, 29]. *Required for Mla6 resistance 3* (*Rar3)* is a novel locus required for *Mla6* function, including H_2_O_2_ accumulation and HR, but segregates independently of both *Mla6* and *Rar1*. *Blufensin1* (*Bln1*) is a negative regulator of PTI signaling [28] and silencing *Bln1* impacts genes associated with basal defense [30]. Overexpression of *Bln1* or its unlinked family member, *Bln2,* increases susceptibility to *Bgh* in compatible interactions*,* while *Barley stripe mosaic virus*-induced gene silencing (BSMV-VIGS) increases resistance [30]. The *mla6* + *bln1* double mutant is susceptible (due to the *mla6* deletion)*,* but PTI-related cellular pathways are deregulated in this background. Each CI 16151-derived barley line has been backcrossed twice to Manchuria with selection followed by at least 4 generations of selfing. Barley lines CI 16151 (*Mla6*), m18982 (*mla6*), m11526 (*rar3*), m19089 (*bln1*), and the m19028 double mutant (*mla6* + *bln1*) were grown with supplemental lighting under temperature-controlled greenhouse conditions. *Bgh* isolate 5874 was propagated on *Hordeum vulgare* cv. Morex in a growth chamber at 18 °C with a 16 h light, 8 h dark day/night cycle.

### Experimental design

Planting, stage of seedlings, inoculation, and sampling of leaf tissue were followed as described previously [29, 63]. Barley tissue was grown in three separate replicates in consecutive weeks. Each of the five genotypes were planted in 20 × 30–cm trays in sterilized potting soil. Each experimental tray consisted of six rows of 12–15 seedling first leaves, with rows randomly assigned to one of the six harvest times in a split-plot design. Within each replicate the five genotypes were infected at 16:00 with a high density of *Bgh* isolate 5874 and harvested at 0, 16, 20, 24, 32, and 48 h after inoculation (HAI) for a total of 90 tissue samples. Total RNA was extracted from *Bgh*-infected barley leaf tissue following the hot (60 °C) phenol/guanidine thiocyanate method described previously [63, 112] and used for RNA-Seq, sRNA-Seq, and PARE [36].

### Small RNA sequencing and data analysis

Small RNA libraries were made with the Illumina TruSeq Small RNA Library kit (Illumina, Inc., San Diego, CA), as per the manufacturer’s protocol. The ninety small RNA Illumina libraries were sequenced on a HiSeq 2500 (Illumina, Inc.) at the Iowa State University DNA Facility in Ames, IA. Reads were quality assessed using the FastQC program version 0.11.3 [113]. Reads were quality filtered and adapters were trimmed using Trimmomatic version 0.33 [114]. Reads were compared with the Rfam database using the Infernal program version 1.1.2 [115] and used to filter tRNAs, rRNAs, snoRNAs and snRNAs from the data. The reads were also filtered using the Triticeae Repeat Sequence Database [116] to remove any known Triticeae-specific repeat sequences. Two programs were used to identify sRNA candidates of interest from barley: miRDeep-P (version 1.3) and ShortStack (version 2.1.0) [33, 34].

### Differential expression of sRNAs

For each time point, we performed a differential expression (DE) analysis, comparing the relative abundance of sRNA reads from the different mutant genotypes to CI 16151 (WT). The sRNA count datasets were normalized and analyzed by using the DESeq2 program package in R [35]. We added 0.5 count units to all read counts and rounded them to the nearest integer to allow use of the DESeq2 normalization method [35]. Reads with 0.9 quantile smaller than a count of 2 are assumed to be expressed at a very low level and were removed from the analysis. The remaining sRNAs were analyzed for DE. The *p*-values were adjusted for multiple testing error using Q-value calculations [117], and sRNAs were filtered for a Q-value of less than 0.05.

### Transcriptome sequencing and analysis

A split-split-plot design was used to run the 90 samples on three Hi-Seq 2500 flow cells. Each replication was run on a separate flow cell with each plant genotype randomly assigned to each of five lanes and 6 barcodes randomly assigned to the 6 time points within each genotype (lane). RNA-sequencing (RNA-Seq) libraries were prepared by the Iowa State University DNA Facility (Ames, Iowa, USA) using the Illumina TruSeq stranded RNA sample preparation kit and were subjected to single-end sequencing (100-bp reads) using the Illumina HiSeq2500 Sequencing System.

The 100 base pair single-end reads were preprocessed using FastQC [113]. Then, the raw reads were processed using Trimmomatic [114]. We (i) cut adapters and Illumina-specific sequences from the reads, (ii) perform a sliding window trimming, cutting once the average quality within the window of 4 base pairs fell below a threshold of 32, (iii) cut bases off the start of a read, if below a threshold quality of 36, (iv) cut bases off the end of a read, if below a threshold quality of 36, and (v) drop the read if it is below a length of 50 base pairs. Then, an additional FastQC [113] check was performed to ensure that any data quality problems were fixed.

Bowtie2 [118] indices were built for the reference genome for barley [119] and *Blumeria* (Ensembl Fungi Assembly EF 1, INSDC Assembly GCA_000151065.1) [8]. The single-end reads were then aligned using the TopHat2 [120] with the “-read-realign-edit-dist” parameter set to 0. This forces TopHat2 to map every read in all the mapping steps (transcriptome, genome, and finally splice variants detected by TopHat2), reporting the best possible alignment found in any of these mapping steps. This may greatly increase the mapping accuracy. This was followed by genome guided Cufflinks version 2.2.1 [121, 122] with the TopHat2 BAM output file as input. Finally, transcripts sequences were extracted with the gffread utility (part of the Cufflinks software) using the GTF file from Cufflinks as input.

For each of the 90 samples, read count estimation was done using RSEM [123] with Trimmomatic [114] trimmed reads as input. Transcript references were built for RSEM along with Bowtie2 indices (rsem-prepare-reference) separately for Blumeria and Barley using respective reference genomes. This was done with the “--gtf” option turned on, this means RSEM assumes that reference file contains the sequence of a genome, and will extract transcript reference sequences using the gene annotations specified in that file. Gene and isoform expression was estimated using “rsem-calculate-expression” with the “--bowtie2” option.

### Parallel analysis of RNA ends (PARE)

PARE libraries were prepared as previously described [36, 124] at the Donald Danforth Plant Science Center in St. Louis, MO and sequenced on a HiSeq 2500 (Illumina, Inc.) at the University of Delaware. Reads were quality assessed using the FastQC program version 0.11.3 [113]. Reads were quality filtered and adapters were trimmed using Trimmomatic version 0.33 [114]. The two PARE analysis programs sPARTA (version 1.21) [31] and CleaveLand (version 4.4) [32] were used independently to identify likely sRNA targets using sRNA sequencing data, the barley transcriptome (ensembl version 38) [53], and PARE sequencing data. PARE validated targets were filtered based on adjusted *p*-values using a 1% false discovery rate along with a PARE category of less than 2 (with sPARTA data).

### PhasiRNA analysis

Identification of *PHAS* loci was completed using methods described previously [125, 126] with minor modification. The sRNA reads were mapped to barley RefSeq1 [53], using bowtie1 [51]. Uniquely mapped reads were chosen for PHAS locus identification. In order to mimic the 3′ overhang, an offset of 2 nucleotides was included for sRNAs that were aligned to the antisense strand of the reference. The reference genome was scanned using a nine-cycle sliding window of 189 bp where each cycle was a user set length of 18 to 26 nt. Windows were reported only when they had at least 10 unique reads, with more than 30% of the reads being the user set length and at least three unique reads falling into the phase registers. Windows with overlapping regions were combined into a larger window. *P*-values for each window was calculated based on the following formula:

$$ p-\mathrm{value}={\sum}_{x=k}^m\frac{\left(\underset{n-x}{\overset{20m}{}}\right)\left(\underset{x}{\overset{m}{}}\right)}{\left(\underset{n}{\overset{21m}{}}\right)} $$

where ‘n’ represents the total number of unique sRNAs of the user set length within the window, ‘m’ was the number of cycles and ‘k’ was the maximum number of unique sRNAs of the user set length falling into one of the possible phase registers. Windows with a *p*-value less than 0.001 were considered as positive PHAS loci.

Phasing score was computed using the methods described by De Paoli and colleagues [127].

$$ \mathrm{Phasing}\ \mathrm{score}=\ln {\left[1+10\frac{\sum \limits_{i=1}^9{P}_i}{1+\sum U}\right]}^{k-2} $$

Where ‘P_i_’ was the total number of reads for all sRNAs of the user set length falling into a given phase within a nine-cycle window, ‘U’ is the total number of reads for all sRNAs of the user let length falling out of the given phase and ‘k’ is the number of phase cycle positions occupied by at least one sRNA of the user set length within the window. The Python/R code and ReadMe file to detect PhasiRNAs is provided at GitHub (https://github.com/Wiselab2/findPhasiRNAs).

## Additional files

Additional file 1: **Figure S1.** Median counts of *Bgh* genome mapped sRNAs for each barley line and time point combination. Reads were mapped to the *Bgh* genome with Bowtie, and median counts from all three replicates for each condition were compared via ANOVA analysis. The null hypothesis was not rejected if the median values are not statistically different with an alpha of 0.05. Standard error bars are shown for each condition. (PDF 24 kb)

Additional file 2: **Figure S2.** PhasiRNA size distributions for genotype-specific phasing. (PDF 382 kb)

Additional file 3: **Table S1.** DE *Bgh*-mapped read details. Differentially expressed reads are detailed including name, genotype and time point differentially expressed, log_2_ fold change, Rfam database membership, and similarity to predicted *Bgh* milRNAs. (XLSX 810 kb)

Additional file 4: **Table S2.** PARE validated predicted miRNAs and *Bgh* genome mapped sRNAs. This table includes details on both the PARE-validated *Bgh* sRNAs as well as their predicted targets including data on PARE validation, sequences, and sRNA target locations. (XLSX 125 kb)

Additional file 5: **Table S3.** PARE validated *Bgh* transcript target annotations. Annotation information for each predicted *Bgh* transcript including ensembl, blastx, interproscan, and literature based categories. (XLSX 28 kb)

Additional file 6: **Table S4.** EKA homolog/hairpin overlap details. Mapping locations, direction of transcript and hairpins, as well as information on overlap type. (XLSX 12 kb)

Additional file 7: **Table S5.** Details of DE barley mapped reads. Differentially expressed barley genome mapped sRNAs details including name, sequence, size, condition DE, and matches to predicted miRNAs and Rfam motifs. (XLSX 287 kb)

Additional file 8: **Table S6.** PARE validated predicted miRNAs and barley genome mapped sRNAs. PARE-validated predicted miRNA and barley genome mapped sRNA information including proposed name, mapping location, predicted transcript targets, and annotations. (XLSX 21 kb)

## Data Availability

The small RNA-Seq datasets supporting the conclusions of this article are available in NCBI’s Gene Expression Omnibus (GEO) under the accession number GSE115992 (https://www.ncbi.nlm.nih.gov/gds/?term=GSE115992). PARE library sequencing is available under GEO accession number GSE116691 (https://www.ncbi.nlm.nih.gov/gds/?term=GSE116691). RNA-Seq data is available as GEO accession number GSE101304 (https://www.ncbi.nlm.nih.gov/gds/?term=GSE101304). The Python/R code and ReadMe file to detect PhasiRNAs is provided at GitHub (https://github.com/Wiselab2/findPhasiRNAs).

## Abbreviations

AVR: Avirulence effector
*Bgh*: *Blumeria graminis* f. sp. *hordei*
CSEP: candidate secreted effector protein
DE: Differential expression
EKA: Effectors homologous to *AVR*_*k1*_ and *AVR*_*a10*_
ETI: Effector-triggered immunity
ETS: Effector-triggered susceptibility
milRNAs: MicroRNA-like
miRNA: Micro RNA
*Mla*: *Mildew resistance locus a*
NLR: Nucleotide-binding, leucine-rich domain proteins / NOD-like Receptors
PAMP: Pathogen-associated molecular pattern
PARE: Parallel Analysis of RNA Ends
phasiRNAs: Phasing small interfering RNAs
RLK: Receptor-like kinase
RNA-Seq: Whole transcriptome sequencing
sRNA-Seq: Small RNA sequencing
TAS: *TRANS ACTING siRNA*
