# Correction to: small RNA discovery in the interaction between barley and the powdery mildew pathogen

**DOI:** 10.1186/s12864-019-6012-7

**Published:** 2019-09-04

**Authors:** Matt Hunt, Sagnik Banerjee, Priyanka Surana, Meiling Liu, Greg Fuerst, Sandra Mathioni, Blake C. Meyers, Dan Nettleton, Roger P. Wise

**Affiliations:** 1 Interdepartmental Genetics & Genomics, Iowa State University, Ames, IA 50011 USA; 2 Department of Plant Pathology & Microbiology, Iowa State University, Ames, IA 50011 USA; 3 Interdepartmental Bioinformatics & Computational Biology, Iowa State University, Ames, IA 50011 USA; 4 Department of Statistics, Iowa State University, Ames, IA 50011 USA; 5 Corn Insects and Crop Genetics Research, USDA-Agricultural Research Service, Iowa State University, Ames, IA 50011 USA; 6 Donald Danforth Plant Science Center, St. Louis, MO 63132 USA; 7 Division of Plant Sciences, University of Missouri – Columbia, 52 Agriculture Lab, Columbia, MO 65211 USA

**Correction to: BMC Genomics**

**https://doi.org/10.1186/s12864-019-5947-z**

Following the publication of the original article [1], the authors noted several typesetting errors which are noted in this Correction article.

In the section “PhasiRNA analysis” there was an error in the first equation in the PDF version of the article. A fraction bar was erroneously introduced between (20 m and n-x), (m and x), and (21 m and n).

The incorrect version was:

$$ \mathrm{p}-\mathrm{value}=\sum \limits_{x=k}^m\frac{\left(\frac{20m}{n-x}\right)\left(\frac{m}{n}\right)}{\left(\frac{21m}{n}\right)} $$

The correct version is:

$$ \mathrm{p}-\mathrm{value}=\sum \limits_{x=k}^m\frac{\left(\begin{array}{c}20m\\ {}n-x\end{array}\right)\left(\begin{array}{c}m\\ {}x\end{array}\right)}{\left(\begin{array}{c}21m\\ {}n\end{array}\right)} $$

Furthermore, due to the formatting of Table 3 there was an error in the formatting of the alignment bars. The correct Table 3 is provided in this Correction article.

**Table 3:** Differentially expressed predicted miRNAs and barley mapped reads with homology to miRBase miRNAs

Predicted miRNA or read	Sequence	miRBase match	Number of predicted barley copies	DE time points (and log_2_ fold changes)	miRBase blastn overlap	Mismatches
DE barley mapped read	TCGGACCAGGCTTCATGCCCC	miR165	NA	*bln1* 16 HAI (-2.55), *bln1* 20 HAI (-2.39), *mla6* 20 HAI (-1.77), *rar3* 20 HAI (-2.14), *bln1* 24 HAI (-2.47), *mla6* 24 HAI (-2.02), *bln1* 48 HAI (-2.41), *mla6* 48 HAI (-1.78)		1
DE barley mapped read	TTCGGACCAGGCTTCCTTCCC	miR166	NA	*mla6* 48 HAI (1.92)		2
DE barley mapped read	TGGGACCAGGCTTCATTCCCC	miR166	NA	*bln1* 20 HAI (-2.24), *rar3* 20 HAI (-1.71)		1
DE barley mapped read	TCGGACCAGGGTTCATTCCCC	miR166	NA	*bln1* 48 HAI (-2.31), *mla6* 48 HAI (-1.80)		1
DE barley mapped read	TTCGGACCAGGCTTCAGTCCC	miR166	NA	*rar3* 48 HAI (-2.10)		2
DE predicted miRNA	ACACAAACCGGGACTAAAG	miR2120	9	*mla6* 20 HAI (1.59)		2
DE predicted miRNA	GTGTTCTCAGGTCGCCCCCGC	miR398	2	*mla6* 32 HAI (2.03)		1
DE predicted miRNA	AGAACAGAGAATGGCGATAGACTC	miR398	1	*mla6* 0 HAI (1.63), *mla6* 20 HAI (1.72), *mla6* 24 HAI (1.66), *mla6* 48 HAI (1.93)		4
DE barley mapped read	TGTGTTCTCAGGTCGCCCCCG	miR398	NA	*mla6* 24 HAI (1.71), *mla6* 32 HAI (2.57)		0
DE predicted miRNA	TCCTGTGCCTGCCTCTTCCAT	miR528	1	*mla6* 20 HAI (1.97), *mla6* 24 HAI (2.27), *mla6* 32 HAI (2.18)		1
DE barley mapped read	TCCTGTGCCTGCCTCTTCCAT	miR528	NA	*mla6* 24 HAI (2.07), *mla6* 32 HAI (2.19)		1
DE barley mapped read	TGGAAGGGGCATGCAGAGGA	miR528	NA	*mla6* 32 HAI (1.86)		0
DE barley mapped read	TGGAAGGGGCATGCAGAGGAG	miR528	NA	*mla6* 16 HAI (2.20), *mla6* 20 HAI (2.40), *mla6* 24 HAI (2.21), *mla6* 32 HAI (2.09)		0
DE barley mapped read	CCTGTGCCTGCCTCTTCCATT	miR528	NA	*mla6* 0 HAI (1.99)		0
DE predicted miRNA	ATTTTGCTTCGTATGTAGACT	none	17	*mla6* 0 HAI (1.97)	none	NA
DE predicted miRNA	TATTAGTTGACAGAGGGAGTA	none	5	*mla6* 48 HAI (-1.77), *mla6- bln1* 48 HAI (-2.44), *bln1* 48 HAI (-2.40)	none	NA
DE predicted miRNA	AACTAGTACTACTCTAATGTGCCT	none	3	*mla6* 0 HAI (-1.07)	none	NA
DE predicted miRNA	GCTTTCATAGCTCAGTTGGTTAGAGCACCCG	none	1	*bln1* 32 HAI (1.64)	none	NA
DE predicted miRNA	AATTTGAACTGTGAAACT	none	1	*mla6* 0 HAI (1.46), *mla6* 20 HAI (1.76), *mla6* 24 HAI (1.56)	none	NA

The publisher would like to apologize to the authors and readers for any inconvenience caused. The original article has been corrected.
