# Supplementary figures and images for: Small RNA discovery in the interaction between barley and the powdery mildew pathogen
Source: BMC Genomics. 2019 Jul 25;20:610. doi: 10.1186/s12864-019-5947-z (PMC6657096; doi:10.1186/s12864-019-5947-z)

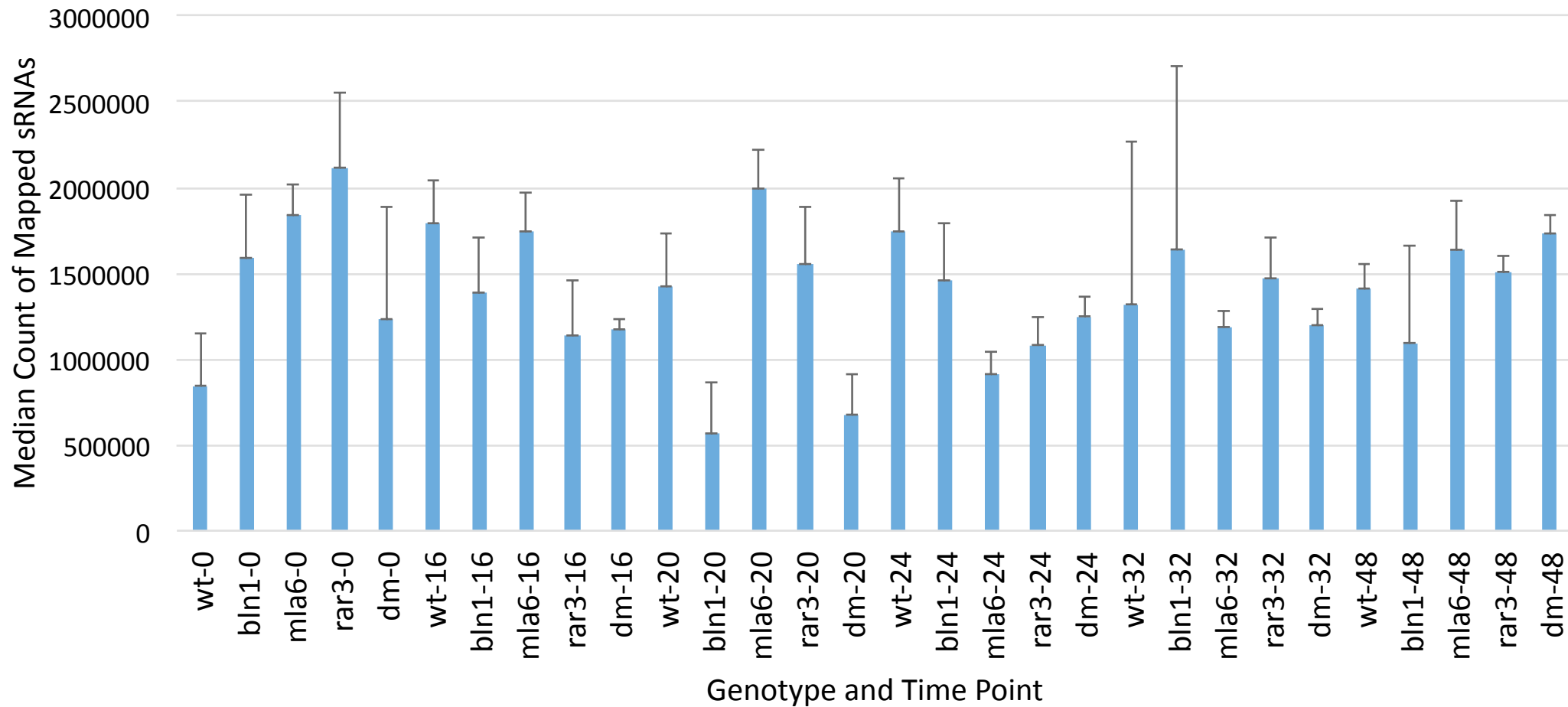

Supplement: Supplementary file 1 — Figure S1. Median counts of Bgh genome mapped sRNAs for each barley line and time point combination. Reads were mapped to the Bgh genome with Bowtie, and median counts from all three replicates for each condition were compared via ANOVA analysis. The null hypothesis was not rejected if the median values are not statistically different with an alpha of 0.05. Standard error bars are shown for each condition. (PDF 24 kb) [file 12864_2019_5947_MOESM1_ESM.pdf]

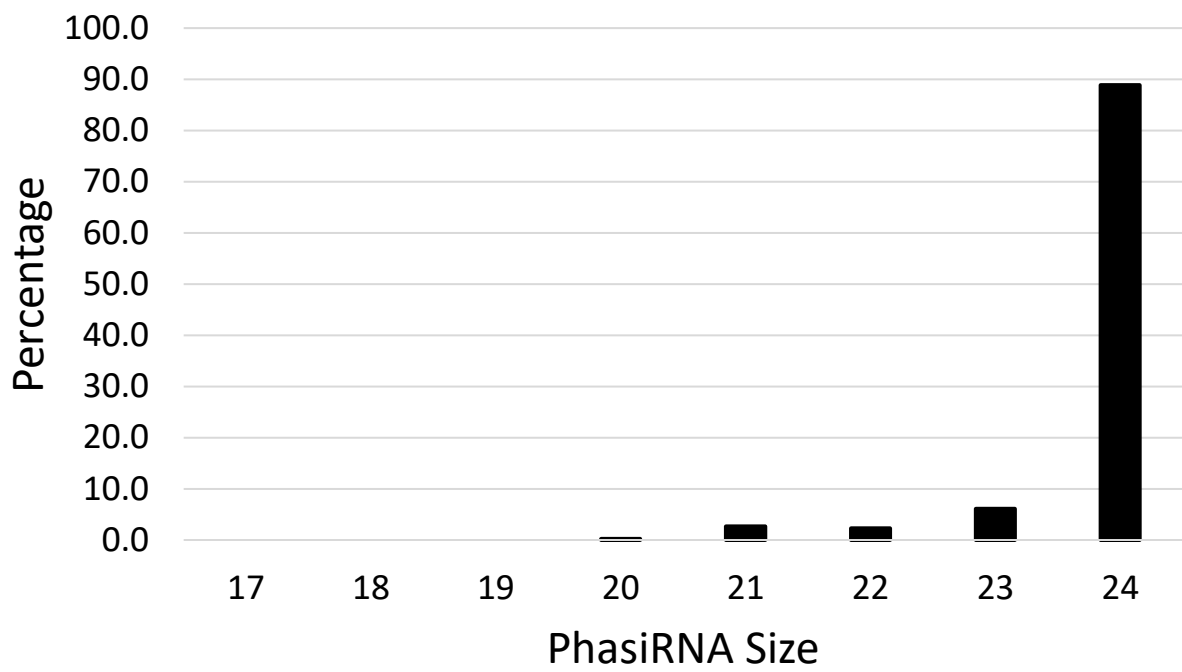

Supplement: Supplementary file 2 — Figure S2. PhasiRNA size distributions for genotype-specific phasing. (PDF 382 kb) [file 12864_2019_5947_MOESM2_ESM.pdf]
